# Biological dispersion in the time domain using finite element method software

**DOI:** 10.1038/s41598-023-49828-1

**Published:** 2023-12-18

**Authors:** Raul Guedert, Daniella L. L. S. Andrade, Guilherme B. Pintarelli, Daniela O. H. Suzuki

**Affiliations:** 1 Department of Electrical and Electronic Engineering, Centre of Technology, Institute of Biomedical Engineering, Federal University of Santa Catarina, Florianopolis, 88040-900 Brazil; 2 Department of Control, Automation and Computer Engineering, Federal University of Santa Catarina, Blumenau, 89036-256 Brazil

**Keywords:** Computational models, Computational biology and bioinformatics, Engineering, Biomedical engineering

## Abstract

Biological tissue exhibits a strong dielectric dispersion from DC to GHz. Implementing biological dispersion in the time domain with commercial finite element method software could help improve engineering analysis of electrical transient phenomena. This article describes the steps required to implement time-domain biological dispersion with commercial finite element method software. The study begins with the presentation of a genetic algorithm to fit the experimental dispersion curve of *Solanum tuberosum* (potato tuber) to multipoles of first-order Debye dispersion. The results show that it is possible to represent the biological dispersion of *S. tuberosum* from 40 Hz to 10 MHz in a 4-pole Debye dispersion. Then, a set of auxiliary differential equations is used to transform the multipole Debye dispersion from the frequency domain to the time domain. The equations are implemented in the commercial software COMSOL Multiphysics. A comparison between the frequency and time domain simulations was used to validate the method. An analysis of the electric current with square-wave pulsed voltage was performed. We found that the computer implementation proposed in this work can describe the biological dispersion and predict the electric current.

Dielectric dispersion is a phenomenon in which dielectric properties change with frequency. The biological tissue is a low-frequency dispersive medium. There are four main dispersion bands in tissues from DC to hundreds of Ghz: $\alpha$, $\beta$, $\delta$, and $\gamma$. The $\alpha$-dispersion usually occurs from DC to 10 kHz and is related to ion polarisation, especially the counterion atmosphere that forms near the surface of charged cells. Some organelles are also associated with $\alpha$-dispersion, such as the sarcoplasmic reticulum in muscle. In addition, the membrane channels dynamics are in this frequency range according to the Hodgkin–Huxley models. The $\beta$-dispersion is usually between 100 kHz and 10 MHz due to Maxwell–Wagner effects caused by the dielectric cell membrane. The $\gamma$-dispersion is related to the relaxation of water molecules and typically occurs around 20 Ghz. The $\delta$-dispersion occurs in the region between the $\beta$- and $\gamma$-dispersion bands due to the polarisation of hydrated proteins and some Maxwell–Wager effects in organelles such as mitochondria and nuclei^1–6^.

Impedance spectroscopy is usually used to study the dielectric properties of biological tissue. Changes in cell structures and tissue composition affect the spectrum. Currently, there are known functions to describe the dielectric dispersion of the tissue, i.e., variation of tissue conductivity and permittivity in the frequency domain. Biological dispersion is often parameterised using the Cole–Cole dispersion, following the extensive tissue parameterisations of Gabriel and colleagues^3–5^ late in the last century. Although the Cole–Cole dispersion is used to characterise the frequency response, it introduces fractional derivatives when transformed to the time domain ^7^. In the absence of simple implementation, biological tissue is often described as a material with constant conductivity in the time domain, which hampers the capacity to interpret electrical stimuli.

Numerical analysis in the frequency domain is widely used in engineering to analyse a steady-state sinusoidal signal. However, the use of the time domain is necessary when transient electrical phenomena are to be studied. Electroporation, for example, is a medical and industrial technology that increases tissue permeability by pulsed electric field (PEF) stimuli^8–10^. Electroporation is a time-dependent transient phenomenon, rendering it unsuitable for study in the frequency domain. Moreover, PEF protocols are usually bursts of square wave pulses that have a broad spectral distribution^11,12^. The implementation of biological dispersion in the time domain could help model its transient and time-dependent electrical phenomena. Ultimately leading to a better understanding of electroporation and its feedback.

There are several works that show numerical solution algorithms to implement a dispersion^13–19^. However, commercial simulation tools are used in some interdisciplinary engineering fields because the learning curve is steep, complex geometries are easier to implement and computer-assisted, and multiphysics analyses can also be performed^20–23^. This work aims to fill the gap in the implementation of biological dispersion in commercial finite element method software in the time domain. To this end, we have performed the parameterisation of biological dispersion data with first-order Debye dispersion using genetic algorithms. The Debye dispersion can then be implemented in the time domain using the auxiliary differential equation method. The proposed implementation was tested using in vitro experiments and simulations with potato tubers (*Solanum tuberosum*) to confirm its feasibility.

## Results

**Figure 1:**
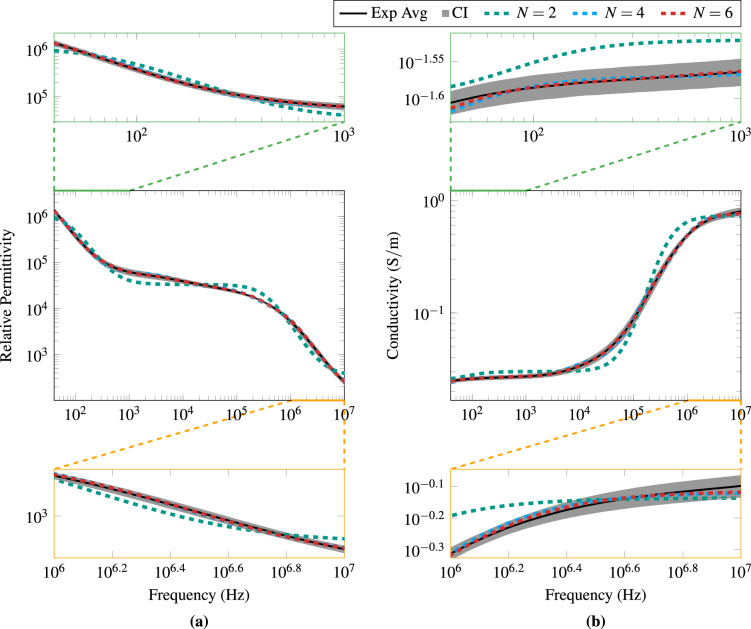
Experimental and parameterised results of permittivity (**a**) and conductivity (**b**) of potato tissue. Exp Avg is the experimental average, CI is the confidence interval (95%). N represents the number of Debye poles used to parameterise the experimental results. The middle images contain the full data. The upper indents are zoomed from 40 Hz to 1 kHz. The lower are zoomed from 1 MHz to 10 MHz.

Table 1 shows the parameterisation values obtained by the genetic algorithm for the Debye multipole model with 2, 4, and 6 poles. Although the parameterisation was performed from 1 to 8 poles, we have shown only three optimisations for the sake of clarity (see the Supplementary Information for the complete set of results). Figure 1 graphically compares the experimental and computer results in terms of the electrical permittivity and conductivity frequency spectrum. We found that the biological dispersion from 40Hz to 10 MHz of potato tissue can be represented with a 4-pole Debye model.

**Table 1:** Parameterisation of potato tissue dispersion with the multipole Debye model with 2, 4, and 6 poles (N).

Parameter	N = 2	N = 4	N = 6
CF min value	1.750	$5.174\times 10^{-2}$	$9.006\times 10^{-3}$
$\varepsilon _{\infty }$	$3.463\times 10^2$	$1.747\times 10^2$	$1.621\times 10^2$
$\sigma _s$	$2.508\times 10^{-2}$	$2.159\times 10^{-2}$	$2.087\times 10^{-2}$
$\Delta \varepsilon _{1}$	$1.104\times 10^6$	$2.251\times 10^6$	$3.198\times 10^6$
$\tau _1$ (s)	$1.932\times 10^{-3}$	$3.783\times 10^{-3}$	$5.067\times 10^{-3}$
$\Delta \varepsilon _{2}$	$3.308\times 10^4$	$2.918\times 10^4$	$3.321\times 10^4$
$\tau _2$ (s)	$4.181\times 10^{-7}$	$2.309\times 10^{-5}$	$3.563\times 10^{-4}$
$\Delta \varepsilon _{3}$		$1.836\times 10^4$	$1.968\times 10^4$
$\tau _3$		$1.005\times 10^{-6}$	$2.495\times 10^{-5}$
$\Delta \varepsilon _{4}$		$1.053\times 10^4$	$1.048\times 10^4$
$\tau _4$ (s)		$1.658\times 10^{-7}$	$3.775\times 10^{-6}$
$\Delta \varepsilon _{5}$			$1.548\times 10^4$
$\tau _5$ (s)			$6.013\times 10^{-7}$
$\Delta \varepsilon _{6}$			$7.628\times 10^3$
$\tau _6$ (s)			$1.403\times 10^{-7}$

CF Min Value is the minimum value reached by the cost function of the genetic algorithm after optimisation.

**Figure 2:**
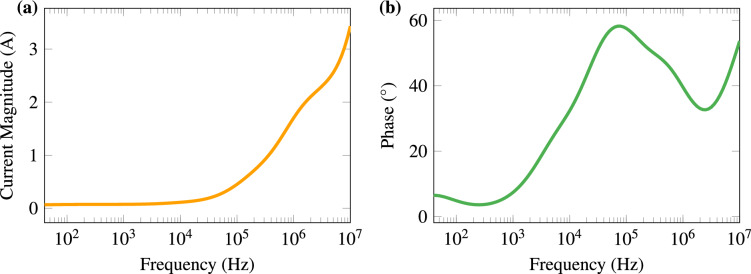
Magnitude (**a**) and phase (**b**) of the electric current in the potato tissue simulated with the 4-pole Debye dispersion.

**Figure 3:**
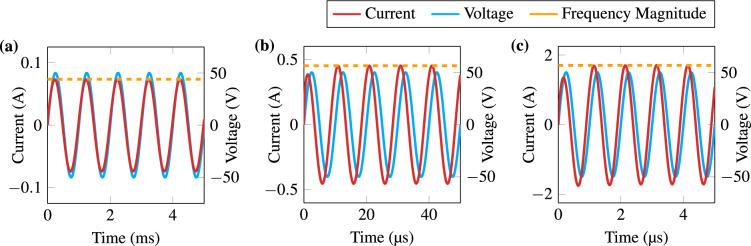
Time domain simulation results of electric current due to an oscillating applied voltage. Input signal frequency of (**a**) 1 kHz, (**b**) 100 kHz, and (**c**) 1 MHz. The Frequency Magnitude is the magnitude found in the frequency domain simulation for the particular input signal frequency.

Figure 2 shows the magnitude and phase of the simulated electric current using the parameterised Debye dispersion with 4 poles. The electric current flow is higher at high frequencies and the current is leading due to the dielectric characteristic. Figure 3 shows the results of the simulated electric currents when applying five periods of an alternating voltage using frequencies of 1 kHz, 100 kHz, and 1 MHz. We also plotted the corresponding current magnitude obtained from the frequency-domain data. To facilitate a comparison between the two types of simulation, we display Table 2 which has the magnitude and phase of 1 kHz, 100 kHz and 1 MHz.

**Table 2:** Numerical results of magnitude and phase for frequency and time domain simulations at 1 kHz, 100 kHz and 1 MHz.

Frequency	Frequency domain		Time domain	
	Magnitude (A)	Phase($^\circ$)	Magnitude (A)	Phase ($^\circ$)
1 kHz	0.0734	7.3949	0.0732	6.0
100 kHz	0.4509	57.494	0.4517	57.6
1 MHz	1.703	39.535	1.706	39.6

**Figure 4:**
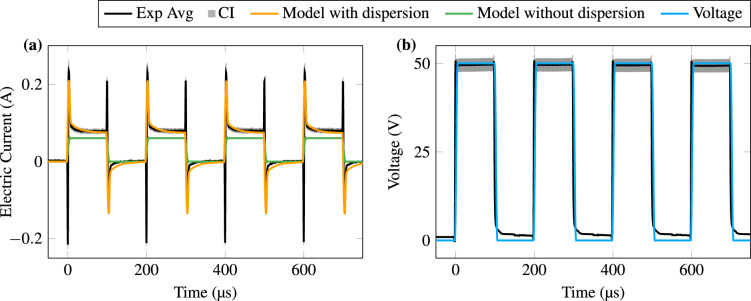
Electric current (**a**) and applied voltage (**b**) for square-wave experiment and simulations. The twelve experimental measurements were summarised as averages (Exp Avg) and confidence interval (CI = 95%). Two computer models were evaluated: with and without the dispersion implementation. The model without the dispersion implementation considers the tissue having a static conductivity. The overshoot of the signal in the opposite direction in PEF transactions is not due to the dispersion itself, but is a common parasitic effect in the experimental setup.

The results of in vitro experiments using a PEF square-wave pulse burst are overlaid in time domain simulations with the proposed dispersion model and a constant conductivity model in Fig. 4. The experimental data were summarised as the average and confidence interval (CI = 95%) of the twelve measurements.

## Discussion

The impedance spectroscopy of the *Solanum tuberosum* shows the presence of the first two biological dispersion bands (Fig. 1). The $\alpha$-dispersion occurs approximately from few Hz to 1 kHz, and the $\beta$-dispersion from 100 kHz to 10 MHz. This is particularly evident in the relative permittivity graph (Fig. 1a), as conductivity is less affected by the $\alpha$-dispersion. On the other hand, conductivity increases more than 10 times due to $\beta$-dispersion, which is reasonable because the plant cell has a cell wall that also acts as a non-conducting layer. This increase in conductivity can also be observed in electric current data from both frequency and time domain simulations (Figs. 2 and 3). Higher frequencies have paths that cross the dielectric cell barrier (cell wall and membrane) in the form of a displacement current.

As for the model parameterisation, the fitting quality is only slightly improved if there are more than 4 Debye poles. Since the time domain implementation increases the computational cost depending on the number of poles, we choose the small number of poles that can fit the curve within the experimental confidence interval. Therefore, the 4-pole model was selected as suitable to satisfy the requirement. It is worth mentioning that a larger number of poles can be expected when the frequency range is increased. Therefore, it is worthwhile to perform the model fitting in the range of interest to obtain the smallest possible number of poles.

To test whether the proposed transformation is indeed feasible, we propose simulations of the same parameterised data in both domains. The simulations in the time and frequency domains are comparable in magnitude and phase when the time domain reaches a sinusoidal steady state. In Fig. 3 one can see that the sinusoidal steady state is reached after one or two periods. Both the magnitude and the phase of the simulated electric current reach similar values regardless of the simulation domain (see Table 2). The magnitude data have up to ± 0.01 error. Phase has up $to \pm 1.5.$ The error between the simulation types may be due to time step definition and numerical approximation. We believe that the convergence of results suggests the feasibility of the proposed domain transformation.

To validate our model, we performed experiments and simulations with a broad spectral signal. The PEF squared pulses used have power mainly below 1 MHz^12^. The 4-pole dispersion model can predict the electric current of the PEF (Fig. 4). The current drop at the top of the pulses indicates that the sample is less conductive at lower frequencies.If the conductivity is assumed to be constant, not only the magnitude of the electric current could be over- or under-estimated based on the conductivity value, but also the waveform deviates. Thus, the model without dispersion cannot meet the 95% CI standard. We observe that there are differences between the model and the experiment during the rise and fall moments (at 0 $\upmu$s and 100 $\upmu$s, and so on). The experiment shows transient overshoots in opposite directions (negative on the rise and positive on the fall). These are common when switching high-frequency signals and could be due to the parasitic capacitance and inductance of the experimental setup. We intentionally did not omit them to make it clear that this is not due to the biological tissue.

## Conclusion

In order to compute the biological dispersion into a time-domain simulation using commercial finite element software, we first had to parameterise the experimental dielectric dispersion with the first-order Debye dispersion using a genetic algorithm. We found that a 4-pole Debye dispersion could describe the experimental curve of *Solanum tuberosum* from 40 Hz to 10 MHz. The first-order dispersion could then be transformed into the time domain using auxiliary differential equations. The suggested methods can be used to represent the frequency response of biological tissue, which is usually measured using impedance spectroscopy. Moreover, by transforming to the time domain, it is possible to predict the experimental electric current of the PEF.

## Methods

We evaluated the equations and performed simulations using COMSOL Multiphysics software (COMSOL Inc., Stockholm, Sweden). COMSOL is a finite element method (FEM) and a computational fluid dynamic (CFD) solver in three domains (static, frequency, and time) with a bundle of built-in physical equation modules. Other commercial software programmes could use the same set of equations and solution methods as COMSOL. We believe that the proposed equations are also suitable for other proprietary software. All simulations presented in this paper were performed with COMSOL Multiphysics version 5.1 (FNL Licence No: 9200073) on a personal computer (AMD Ryzen 3800X processor, 8 cores, 16 threads at 4.2 GHz, AMD Radeon 5700XT graphics card, 16 GB RAM) running Windows 11.

We tested the implementation with two sets of simulations. First, we simulated the parameterised dispersion in the frequency domain and compared the results with sinusoidal signals in the time domain. Then we performed an experiment with square-wave pulses (since they have a wide spectral distribution) and compared them with a simulation in the time domain. The geometry for both tests followed that used in vitro.

To solve electrodynamics problems at low frequency, we can use the principle of charge conservation and solve the equation of continuity shown in Eq. (1).

1 $$\begin{aligned} {\vec {\nabla }} \cdot \vec {J} = - \frac{\partial \rho }{\partial t} \end{aligned}$$

where $\rho$ is the total charge density and $\vec {J}$ is the electric current density which components are given in Eq. (2).

2 $$\begin{aligned} \vec {J}(t) = \sigma _s \vec {E}(t) + \varepsilon _0 \frac{\partial \varepsilon _r \vec {E}(t)}{\partial t} + \vec {J}_e(t) \end{aligned}$$

$\sigma _s$ is the initial conductivity, $\vec {E}(t)$ is the electric field, $\vec {J}_e(t)$ is an arbitrary external current density, $\varepsilon _0$ is the dielectric constant of the vacuum, and $\varepsilon _r$ is the relative permittivity of the material.

In studies in the frequency domain, this set of equations is transformed using the Fourier transform, as represented in the Eq. (3).

3 $$\begin{aligned} \vec {J}(\omega ) = \sigma _s \vec {E}(\omega ) + j\omega \varepsilon _0 \varepsilon _r \vec {E}(\omega ) + \vec {J}_e(\omega ) \end{aligned}$$

### In vitro samples

We bought potato tubers (*Solanum tuberosum*) from a local producer (Caore Orgânicos, Rio Grande do Sul, Brazil). The producer is certified for organically grown products by the Participatory Conformity Assessment Organisation (OPAC), registered with the Brazilian Ministry of Agriculture, Livestock and Food Supply (MAPA). Each sample weighed approximately 100 g and had no sprouts. The potatoes were washed and cut in half with a stainless steel knife. We made a cylindrical incision in each half with a stainless steel seed remover of 18.50 mm diameter. Then we cut the cylindrical fragment into 5 mm (± 0.2 mm) high samples. The final samples resulted in cylindrical fragments 5 mm height with a diameter of 18.50 mm. The samples were wrapped in paper towels to prevent denaturation and reduce oxidation before experiments. The laboratory temperature was 20 $^\circ$C. The cylindrical samples were used in the following experiments.

Ten potato samples were subjected to impedance spectroscopy analysis using the Agilent 4294A Precision Impedance Analyser (Agilent Technologies, California, USA). Each sample was placed between two square gold plates (20 mm$\times$20 mm) and carefully fixed with a spring clamp. The impedance analyser swept the frequency from 40 Hz to 10 MHz (20 points per decade) and recorded the admittance.

In the square-wave pulse experiment, twelve samples were subjected to a sequence of four pulses 100 $\upmu$s long at 50 V with each pulse repeated every 200 $\upmu$s. Each sample was placed between the electrodes and carefully fixed with a spring clamp, received one square-wave pulse burst and was then replaced. Current and voltage were digitally stored using a Tektronix DPO2012B oscilloscope (Tektronix Inc., www.tek.com) with Tektronix TPP0100 and Tektronix A622 voltage and current probes, respectively.

### Describing a dielectric dispersion using first-order Debye dispersion

The relative permittivity of biological tissue is generally described using the Cole–Cole dispersion presented in Eq. (4).

4 $$\begin{aligned} {\varepsilon }^{*}_r (\omega ) = \frac{\sigma _s}{j\omega \varepsilon _0} + \varepsilon _\infty + \sum _{k=1}^{N} \frac{\Delta \varepsilon _k}{1 + \left( j\omega \tau _k \right) ^{1 - \lambda _k}} \end{aligned}$$

where ${\varepsilon }^{*} (\omega )$ is the complex permittivity, $\sigma _s$ is the static conductivity, $\varepsilon _\infty$ is the high frequency permittivity, *k* represents the pole of the Cole–Cole dispersion, $\Delta \varepsilon _k$ is the increase in the permittivity due to the dispersion pole, $j\omega$ is the complex angle, $\tau _k$ is the relaxation time of the pole, and $\lambda _k$ is an empirical factor to fit the curve, varying between 0 and 1. It should be noted that the Cole–Cole dispersion already includes static conductivity in its definition, which is made possible by factorising the electric field ($\vec {E}(\omega )$) in Eq. (3) (see Supplementary Information for this factorisation).

If one attempts to substitute Eqs. (4) into (3) and perform the Fourier inverse transform, fractional derivatives are obtained in the time domain for $0< \lambda _k < 1$. The lack of a direct implementation in the time domain is a well-known limitation of the Cole–Cole dispersion. However, any continuous dispersion can be represented as a linear combination of first-order dispersions^24^. The biological dispersion could then be described as a multipole of the first-order Debye dispersion. Transformation of the Debye dispersion from the frequency domain to the time domain can be performed with different methods^13–19^. The Debye dispersion is given in Eq. (5).

5 $$\begin{aligned} {\varepsilon }^{*}_r (\omega ) = \frac{\sigma _s}{j\omega \varepsilon _0} + \varepsilon _\infty + \sum _{k=1}^{N} \frac{\Delta \varepsilon _k}{1 + \left( j\omega \tau _k \right) } \end{aligned}$$

We measure the admittance ($Y(\omega )$) of the material in the experimental impedance analysis, which is composed of the conductance ($G(\omega )$) and susceptance ($B(\omega )$), i.e., $Y = G + jB$. Conductance is geometrically related to conductivity, whereas susceptance is geometrically related to permittivity. We can relate both the susceptance (Eq. 6) and the conductance (Eq. 7) of the experimental data as a function of the real and imaginary parts of the Debye dispersion shown in Eq. (5) (we leave the derivation of the equations in the Supplementary Information). Then we can use these functions to perform the parameterisation.

6 $$\begin{aligned} \Re \left( {\varepsilon }_{r}^{*} (\omega ) \right) = \varepsilon _{r}(\omega ) = \frac{B(\omega )}{\omega \varepsilon _0} \frac{l}{S} \end{aligned}$$

7 $$\begin{aligned} \Im \left( {\varepsilon }_{r}^{*} (\omega ) \right) = - \frac{\sigma (\omega )}{\omega \varepsilon _{0}} = - \frac{G(\omega )}{\omega \varepsilon _{0}} \frac{l}{S} \end{aligned}$$

where *l* and *S* are the length and electrode contact area of the sample, respectively.

There are several ways to fit the experimental data to a Debye dispersion representation^25–27^. We have found the best results fitting the model with genetic algorithms^26,28^. Genetic algorithms are a powerful tool for finding the global minimum of functions. In model fitting, genetic algorithms are used to find the minimum of a cost function that quantifies the fit of the model. An input parameter interval should be set to support the convergence of the genetic algorithm and avoid results without physical meaning (e.g., $\tau _k < 0$ or $\varepsilon _\infty < 1$). We used the recommendations of Clegg and Robinson^28^ on the parameter intervals for the Debye dispersion. The authors also recommend using the logarithmic scale because the dispersion parameter has a wide range of values on the linear scale. The parameter intervals are defined as follows: $\log _{10}{\Delta \varepsilon _k} \in (-3; 8)$, $\log _{10}{\tau _k} \in (-12; 1)$, $\log _{10}{\sigma _s} \in (-4; 0)$, and $\log _{10}{\varepsilon _\infty } \ge 0$.

The cost function was defined according to Krewer and O’Halloran^26^. The error between the experimental data and its representation by the Debye dispersion is calculated by comparing the real and imaginary parts of the two functions. Again, the logarithmic scale is required to avoid fitting bias. Note that here we are using the frequency in Hertz instead of the angular frequency ($\omega = 2 \pi f$).

8 $$\begin{aligned} C_{f} = \sum _{f={40}\textrm{Hz}}^{f={10}~\textrm{MHz}} \left( \log _{10}{E_r(f)} - \log _{10}{D_r(f)} \right) ^2 + \left( \log _{10}{E_i(f)} - \log _{10}{D_i(f)} \right) ^2 \end{aligned}$$

where $E_r(f)$ and $E_i(f)$ are the average real and imaginary parts of the experimental data obtained from Eqs. (6) and (7). $D_r(f)$ and $D_i(f)$ are the real and imaginary parts of the multipole Debye dispersion. The sum includes the entire measured frequency spectrum.

We performed parameterisation with 1 to 8 Debye poles. The genetic algorithm was configured as follows. The population size was 1000. The tournament size was 20. The probability of mutation was 10%. The maximum number of generations was 2000. The algorithm was implemented using MATLAB 2018a (MathWorks, Massachusetts, USA). The code is available in the Supplementary Information.

### Transforming the Debye dispersion into the time domain

We found that the three common methods for transforming dispersion from frequency to time domain are recursive convolution (RC), the auxiliary differential equation (ADE) method, and Z-transform^13–19^. Although the Z-transform method is often described as simple to implement and effective in custom algorithms, its implementation in COMSOL becomes complex because it requires discrete operations that are neither performant nor trivial to implement in COMSOL. On the other hand, COMSOL provides the user with the ability to easily incorporate ordinary differential equations (ODE) into the simulation. For this reason, we used the ADE method to transform the dispersion from frequency to time domain.

To carry out the transformation using the ADE method^13^, we first add a single-pole Debye dispersion presented in Eq. (5) into the low-frequency Maxwell–Ampère law given in Eq. (3). After some algebraic simplifications, one finds

9 $$\begin{aligned} \vec {J}(\omega ) = \sigma _s \vec {E}(\omega ) + j\omega \varepsilon _0\varepsilon _\infty \vec {E}(\omega ) + j\omega \varepsilon _0 \frac{\Delta \varepsilon }{1 + \left( j\omega \tau \right) }\vec {E}(\omega ) \end{aligned}$$

The Debye pole component can now be defined as an arbitrary current density given by

10 $$\begin{aligned} \vec {J}_e(\omega ) = j\omega \varepsilon _0 \frac{\Delta \varepsilon }{1 + \left( j\omega \tau \right) }\vec {E}(\omega ) \end{aligned}$$

and multiplying both sides of the equation by $1 + \left( j\omega \tau \right)$, one finds that

11 $$\begin{aligned} \vec {J}_e(\omega ) + j\omega \tau \vec {J}_e(\omega ) = j\omega \varepsilon _0 \Delta \varepsilon \vec {E}(\omega ) \end{aligned}$$

So, one can perform the Fourier inverse transform, which leads to

12 $$\begin{aligned} \vec {J}_e(t) + \tau \frac{\partial \vec {J}_e(t)}{\partial t} = \varepsilon _0\Delta \varepsilon \frac{\partial \vec {E}(t)}{\partial t} \end{aligned}$$

The full current density can also be transformed, leading to the following equation.

13 $$\begin{aligned} \vec {J}(t) = \sigma _s \vec {E}(t) + \varepsilon _0\varepsilon _\infty \frac{\partial \vec {E}(t)}{\partial t} + \vec {J}_e(t) \end{aligned}$$

Equation (13) is the low-frequency time-domain Maxwell–Ampère law. Note that $\vec {J}_e(t)$ is calculated using the ADE given in the Eq. (12). The ADE can be optimised to reduce dependence on the electric field derivative over time. The COMSOL documentation helps us to define an auxiliary electric field ($\vec {e}$) to improve $\vec {J}_e$ computation.

14 $$\begin{aligned} \vec {J}_e(t) \equiv \varepsilon _0\Delta \varepsilon \frac{\partial \vec {e}(t)}{\partial t} \end{aligned}$$

Replace the definition of Eqs. (14) into (12).

15 $$\begin{aligned} \varepsilon _0\Delta \varepsilon \frac{\partial \vec {e} (t)}{\partial t} + \tau \varepsilon _0\Delta \varepsilon \frac{\partial ^2 \vec {e}(t) }{\partial t^2} = \varepsilon _0\Delta \varepsilon \frac{\partial \vec {E}(t)}{\partial t} \end{aligned}$$

The equation could then be simplified by the common term $\left( \varepsilon _0\Delta \varepsilon \right)$ and integrated once in time, assuming that the initial fields are zero (thus the integration constant is zero). This leads to the ordinary differential equation (ODE).

16 $$\begin{aligned} \tau \frac{\partial \vec {e}(t)}{\partial t} =\vec {E}(t) - \vec {e}(t) \end{aligned}$$

Let us return to the definition of the auxiliary electric field and substitute the relation found in Eq. (16).

17 $$\begin{aligned} \vec {J}_e(t) = \frac{\varepsilon _0\Delta \varepsilon }{\tau } \left( \vec {E}(t) - \vec {e}(t) \right) \end{aligned}$$

Then the Debye dispersion can be introduced into the time domain as an arbitrary current density given by Eq. (17), which is calculated using the ODE represented in Eq. (16). Although only one Debye pole was used in the previous calculations, it is not difficult to extend the equations to a multipole equation. Thus, the following set of equations describes a multipole Debye dispersion in the time domain.

18 $$\begin{aligned} \vec {J}(t) = \sigma _s \vec {E}(t) + \varepsilon _0\varepsilon _\infty \frac{\partial \vec {E}(t)}{\partial t} + \sum _{k=1}^{N} \vec {J}_{e_k}(t) \end{aligned}$$

19 $$\begin{aligned} \vec {J}_{e_k}(t) = \frac{\varepsilon _0\Delta \varepsilon _k}{\tau _k} \left( \vec {E}(t) - \vec {e_k}(t) \right) \end{aligned}$$

20 $$\begin{aligned} \tau _k \frac{\partial \vec {e_k}(t)}{\partial t} = \vec {E}(t) - \vec {e_k}(t) \end{aligned}$$

where *k* represents a Debye dispersion pole.

Domain Ordinary Differential Equation (DODE) physics module can be used to incorporate each ODE into COMSOL. It should be noted that each axis of the geometric problem must be considered. In the case of a three-dimensional (3D) model in Cartesian axes, the Eq. (20) is included with the matrix equation below.

21 $$\begin{aligned} \begin{bmatrix} \tau _k &{}\quad 0 &{}\quad 0 \\ 0 &{}\quad \tau _k &{}\quad 0 \\ 0 &{}\quad 0 &{}\quad \tau _k \end{bmatrix}\frac{\partial }{\partial t}\begin{bmatrix}e_{k_x} \\ e_{k_y} \\ e_{k_z} \end{bmatrix} = \begin{bmatrix}E_x \\ E_y \\ E_z \end{bmatrix} - \begin{bmatrix}e_{k_x} \\ e_{k_y} \\ e_{k_z} \end{bmatrix} \end{aligned}$$

Note that each Debye pole includes three ODE when simulating a three-dimensional (3D) geometry. One should reduce the number of poles as much as possible to reduce the computation time, especially for complex geometries.

### Computer finite element study

The geometry was created using the COMSOL Geometry tool. We used the symmetry of the sample to simulate it with an axisymmetric 2D geometry. In the axisymmetric 2D geometry, the sample cylinder is formed by a rotated rectangle 9.25 mm long and 5 mm high. The inner boundary was used to perform the rotation of axis symmetry. The upper and lower boundaries were considered as terminal and ground, respectively (Dirichlet boundary condition). The outer boundary was considered as insulating (Neumman boundary condition). The meshes were generated using the COMSOL Mesh Generation tool at fine resolution, resulting in 524 domain elements. The dielectric properties obtained in the parameterisation step were implemented in the geometry. The Eq. (5) can be implemented directly in the frequency domain. In the time domain, the implementation followed Eqs. (18)–(20). We used a dynamic time step with the intermediate generalised alpha method when working with the time domain. The intermediate generalised alpha method allows the solver to decrease the time step to improve convergence. The maximum time step is indicated for each study in the following.

For comparison of the two domain-type simulations, we set the voltage magnitude to 50 V. In the frequency domain, the frequency range was varied between 40 Hz and 10 MHz, 100 points per decade. In the time domain, we simulated five periods of sine waves with frequencies of 1 kHz, 100 kHz, and 1 MHz. The maximum time step was set to one hundredth of the signal period. We then compared the magnitude and phase of the electric current between the two domain simulations to test the validity of the domain transformation.

In the square-wave pulse experiment, the input signal followed the experimental. The pulse transition time was set to 8$\upmu$s. For comparison purposes, we also ran a simulation with the dispersion removed and only the static conductivity ($\sigma _s$) of Table 1 considered. The maximum time step was established at 0.1 $\upmu$s in transition regions and at 1 $\upmu$s otherwise.

### Supplementary Information

Supplementary Information.

## Data Availability

The datasets generated and analysed during the current study are available in the figshare repository, www.doi.org/10.6084/m9.figshare.23895927.
