# Supplementary material for: Biological dispersion in the time domain using finite element method software
Source: Sci Rep. 2023 Dec 18;13:22868. doi: 10.1038/s41598-023-49828-1 (PMC10739869; doi:10.1038/s41598-023-49828-1)
Supplement: Supplementary file 1 — Supplementary Information. [file 41598_2023_49828_MOESM1_ESM.pdf]

Biological dispersion in the time domain using finite element method software

Raul Guedert[1,*], Daniella L. L. S. Andrade[1], Guilherme B. Pintarelli[2], and Daniela O. H. Suzuki[1]

[1]Federal University of Santa Catarina, Institute of Biomedical Engineering, Florianopolis, 88040-900, Brazil
[2]Department of Control and Automation Engineering, Federal University of Santa Catarina, Blumenau, 89036-256, Brazil.
[*]raulguedert@gmail.com

SUPPLEMENTARY INFORMATION

The datasets generated and analysed during the current study are available in the figshare repository, www.doi.org/10.6084/m9.figshare.23895927.

Contents

S.1 Conductivity and Relative Permittivity Derivation . . . 1
S.2 Experimental Setup . . . 3
S.3 Biological Dispersion Parametrization Results . . . 4
S.4 Genetic Algorithm Code . . . 7

S.1 Conductivity and Relative Permittivity Derivation

Equation (S1) presents the Maxwell-Ampère law in the frequency domain.

$$\vec{J}(\omega) = \sigma_s \vec{E}(\omega) + j\omega\varepsilon_0\varepsilon_r\vec{E}(\omega) + \vec{J}_e(\omega) \tag{S1}$$

We can factor the electric field ($\vec{E}(\omega)$), which gives us.

$$\vec{J}(\omega) = j\omega\varepsilon_0 \overbrace{\left(\frac{\sigma_s}{j\omega\varepsilon_0} + \varepsilon_r\right)}^{\varepsilon_r^*(\omega)} \vec{E}(\omega) + \vec{J}_e(\omega) \tag{S2}$$

The multipole Debye model is given in (S3).

$$\varepsilon_r^*(\omega) = \frac{\sigma_s}{j\omega\varepsilon_0} + \varepsilon_\infty + \sum_{k=1}^{N} \frac{\Delta\varepsilon_k}{1 + (j\omega\tau_k)} \tag{S3}$$

The Debye model can be discriminated into real and imaginary parts according to the definitions in equation (S4).

$$\varepsilon_r^*(\omega) = \varepsilon^{'}(\omega) - j\varepsilon^{''}(\omega) \tag{S4}$$

where $\varepsilon^{'}(\omega)$ and $\varepsilon^{''}(\omega)$ are given by

$$\begin{aligned} \varepsilon^{'}(\omega) &= \Re\left(\varepsilon_r^*(\omega)\right) \\ \varepsilon^{''}(\omega) &= -\Im\left(\varepsilon_r^*(\omega)\right) \end{aligned} \tag{S5}$$

Now we can substitute the relation of equation (S4) into the Maxwell-Ampère law represented in equation (S2), which gives us the following.

$$\vec{J}(\omega) = j\omega\varepsilon_0 \left(\varepsilon^{'}(\omega) - j\varepsilon^{''}(\omega)\right)\vec{E}(\omega) + \vec{J}_e(\omega) \tag{S6}$$

After some algebraic simplification, one finds the following.

$$\vec{J}(\omega) = \overbrace{\omega\varepsilon_0\varepsilon^{''}(\omega)}^{\sigma(\omega)}\vec{E}(\omega) + j\omega\varepsilon_0\overbrace{\varepsilon^{'}(\omega)}^{\varepsilon_r(\omega)}\vec{E}(\omega) + \vec{J}_e(\omega) \tag{S7}$$

By comparing equations (S1) and (S7), we can determine conductivity and relative permittivity.

$$\sigma(\omega) = \omega\varepsilon_0\varepsilon^{''}(\omega) = -\omega\varepsilon_0\Im\left(\varepsilon_r^*(\omega)\right) = \sigma_s + \omega^2\varepsilon_0\sum_{k=1}^{N}\tau_k\frac{\Delta\varepsilon_k}{1+(\omega\tau_k)^2} \tag{S8}$$

$$\varepsilon_r(\omega) = \varepsilon^{'}(\omega) = \Re\left(\varepsilon_r^*(\omega)\right) = \varepsilon_\infty + \sum_{k=1}^{N}\frac{\Delta\varepsilon_k}{1+(\omega\tau_k)^2} \tag{S9}$$

The relation between conductance (G) and susceptance (B) with conductivity and relative permittivity is given by equations (S10) and (S11), respectively.

$$\sigma(\omega) = G(\omega)\frac{l}{S} \tag{S10}$$

$$\varepsilon_r(\omega) = \frac{B(\omega)}{\omega\varepsilon_0}\frac{l}{S} \tag{S11}$$

where l and S are the length and area of the sample, respectively.

By combining equations (S8) and (S9) with equations (S10) and (S11), one finds the following relation.

$$\Im\left(\varepsilon_r^*(\omega)\right) = -\frac{\sigma(\omega)}{\omega\varepsilon_0} = -\frac{G(\omega)}{\omega\varepsilon_0}\frac{l}{S} \tag{S12}$$

$$\Re\left(\varepsilon_r^*(\omega)\right) = \varepsilon_r(\omega) = \frac{B(\omega)}{\omega\varepsilon_0}\frac{l}{S} \tag{S13}$$

S.2 Experimental Setup

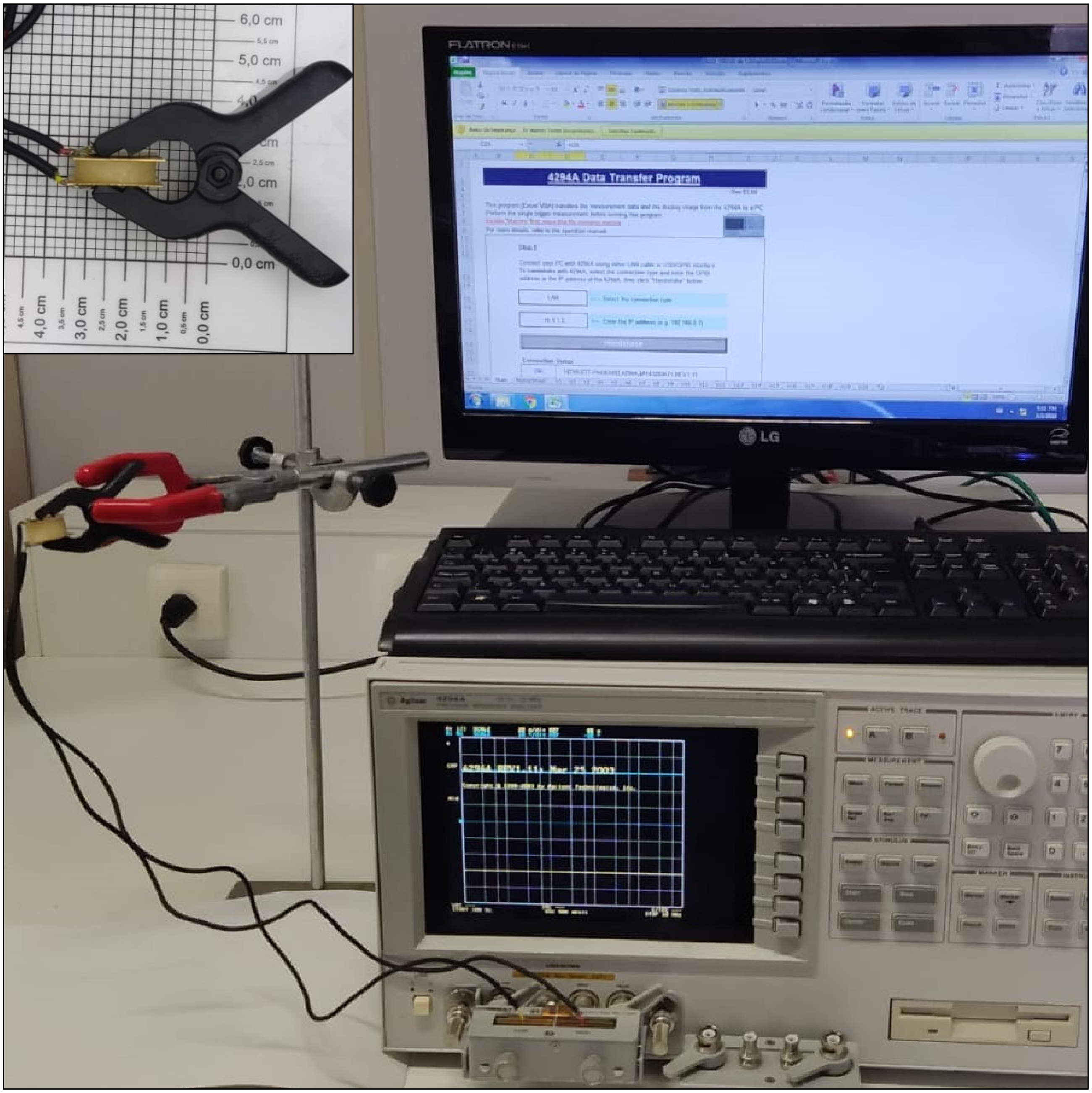

Figure S1. Experimental setup for the dielectric measurement of potato tuber samples. The electrodes can be seen in detail at the top left.

S.3 Biological Dispersion Parametrization Results

Parameter	N = 1	N = 2	N = 3	N = 4
CF Min Value	6.245×10^{1}	1.750	2.908×10^{-1}	5.174×10^{-2}
ε_∞	1.093×10^{3}	3.463×10^{2}	2.352×10^{2}	1.747×10^{2}
σ_s	3.015×10^{-2}	2.508×10^{-2}	2.208×10^{-2}	2.159×10^{-2}
$\Delta\varepsilon_1$	5.041×10^{2}	1.104×10^{6}	1.753×10^{6}	2.251×10^{6}
τ_1 (s)	5.214×10^{-6}	1.932×10^{-3}	3.071×10^{-3}	3.783×10^{-3}
$\Delta\varepsilon_2$		3.308×10^{4}	3.004×10^{4}	2.918×10^{4}
τ_2 (s)		4.181×10^{-7}	6.431×10^{-6}	2.309×10^{-5}
$\Delta\varepsilon_3$			1.919×10^{4}	1.836×10^{4}
τ_3			2.530×10^{-7}	1.005×10^{-6}
$\Delta\varepsilon_4$				1.053×10^{4}
τ_4 (s)				1.658×10^{-7}

Table S1. Parameterization of potato tissue dispersion with the multipole Debye model with 1, 2, 3, and 4 poles. CF Min Value is the minimum value reached by the cost function of the genetic algorithm after optimization.

Parameter	N = 5	N = 6	N = 7	N = 8
CF Min Value	2.287×10^{-2}	9.006×10^{-3}	5.583×10^{-3}	4.358×10^{-3}
ε_∞	1.765×10^{2}	1.621×10^{2}	1.495×10^{2}	1.354×10^{2}
σ_s	2.124×10^{-2}	2.087×10^{-2}	2.061×10^{-2}	2.074×10^{-2}
$\Delta\varepsilon_1$	2.683×10^{6}	3.198×10^{6}	3.604×10^{6}	3.129×10^{6}
τ_1 (s)	4.407×10^{-3}	5.067×10^{-3}	5.543×10^{-3}	5.183×10^{-3}
$\Delta\varepsilon_2$	2.458×10^{4}	3.321×10^{4}	1.863×10^{4}	2.983×10^{5}
τ_2 (s)	1.376×10^{-4}	3.563×10^{-4}	8.438×10^{-4}	7.310×10^{-3}
$\Delta\varepsilon_3$	1.950×10^{4}	1.968×10^{4}	2.868×10^{4}	3.674×10^{4}
τ_3	1.097×10^{-5}	2.495×10^{-5}	3.763×10^{-4}	3.873×10^{-4}
$\Delta\varepsilon_4$	1.654×10^{4}	1.048×10^{4}	1.993×10^{4}	2.116×10^{4}
τ_4 (s)	8.483×10^{-7}	3.775×10^{-6}	2.733×10^{-5}	2.340×10^{-5}
$\Delta\varepsilon_5$	1.005×10^{4}	1.548×10^{4}	1.182×10^{4}	1.243×10^{4}
τ_5 (s)	1.636×10^{-7}	6.013×10^{-7}	3.684×10^{-6}	2.509×10^{-6}
$\Delta\varepsilon_6$		7.628×10^{3}	1.649×10^{4}	1.607×10^{4}
τ_6 (s)		1.403×10^{-7}	5.100×10^{-7}	3.869×10^{-7}
$\Delta\varepsilon_7$			5.830×10^{3}	6.852×10^{2}
τ_7 (s)			1.215×10^{-7}	1.205×10^{-7}
$\Delta\varepsilon_8$				2.993×10^{3}
τ_8 (s)				9.330×10^{-8}

Table S2. Parameterization of potato tissue dispersion with the multipole Debye model with 5, 6, 7, and 8 poles. CF Min Value is the minimum value reached by the cost function of the genetic algorithm after optimization.

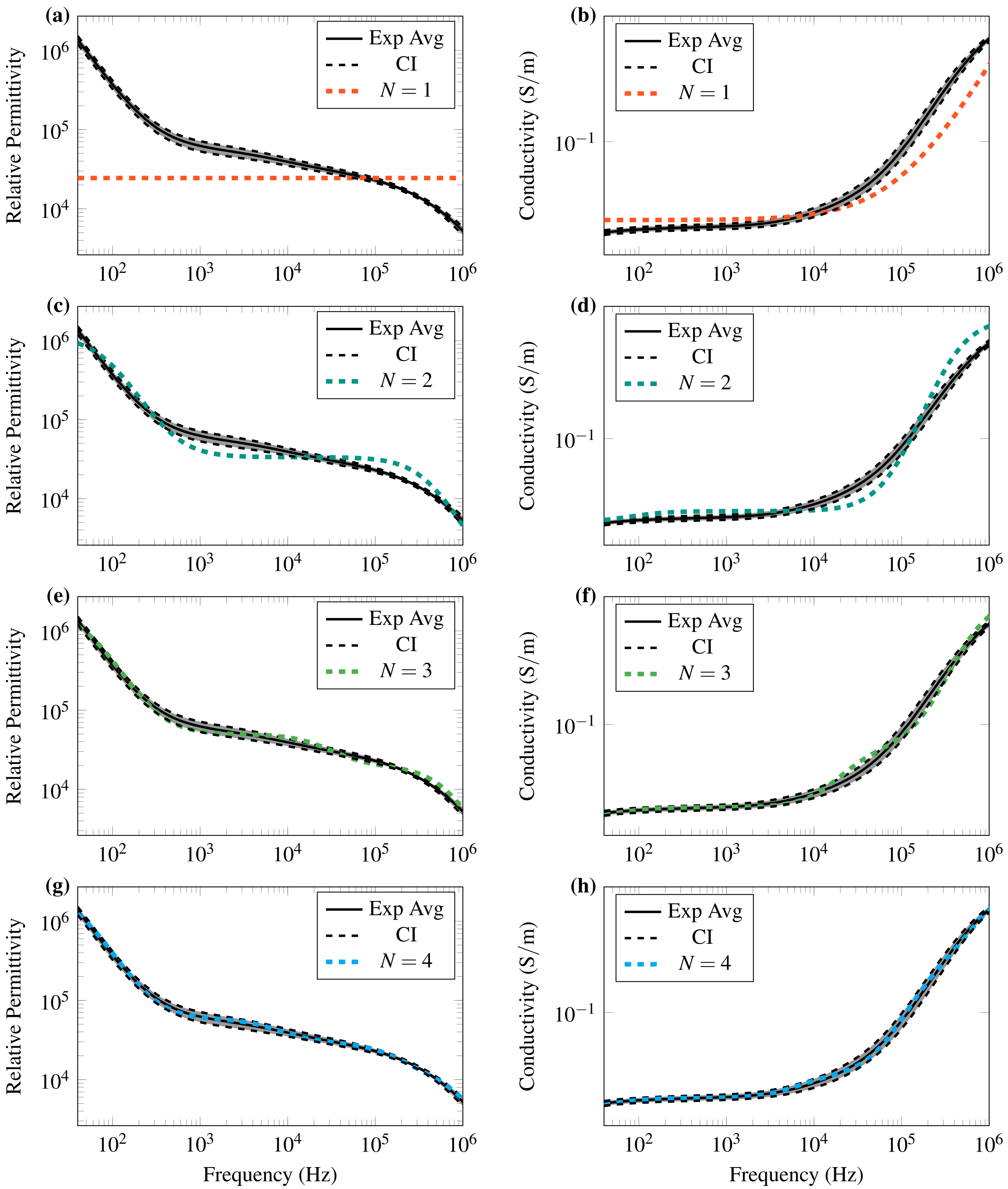

Figure S2. Experimental and parameterized results of **(a, b, c, d)** permittivity and **(b, d, f, h)** conductivity of potato tissue. Exp Avg is the experimental average, CI is the confidence interval (95%). N represents the number of Debye poles used to parameterize the experimental results. (**a, b**) 1 pole; **(c, d)** 2 poles; **(e, f)** 3 poles; **(g, h)** 4 poles.

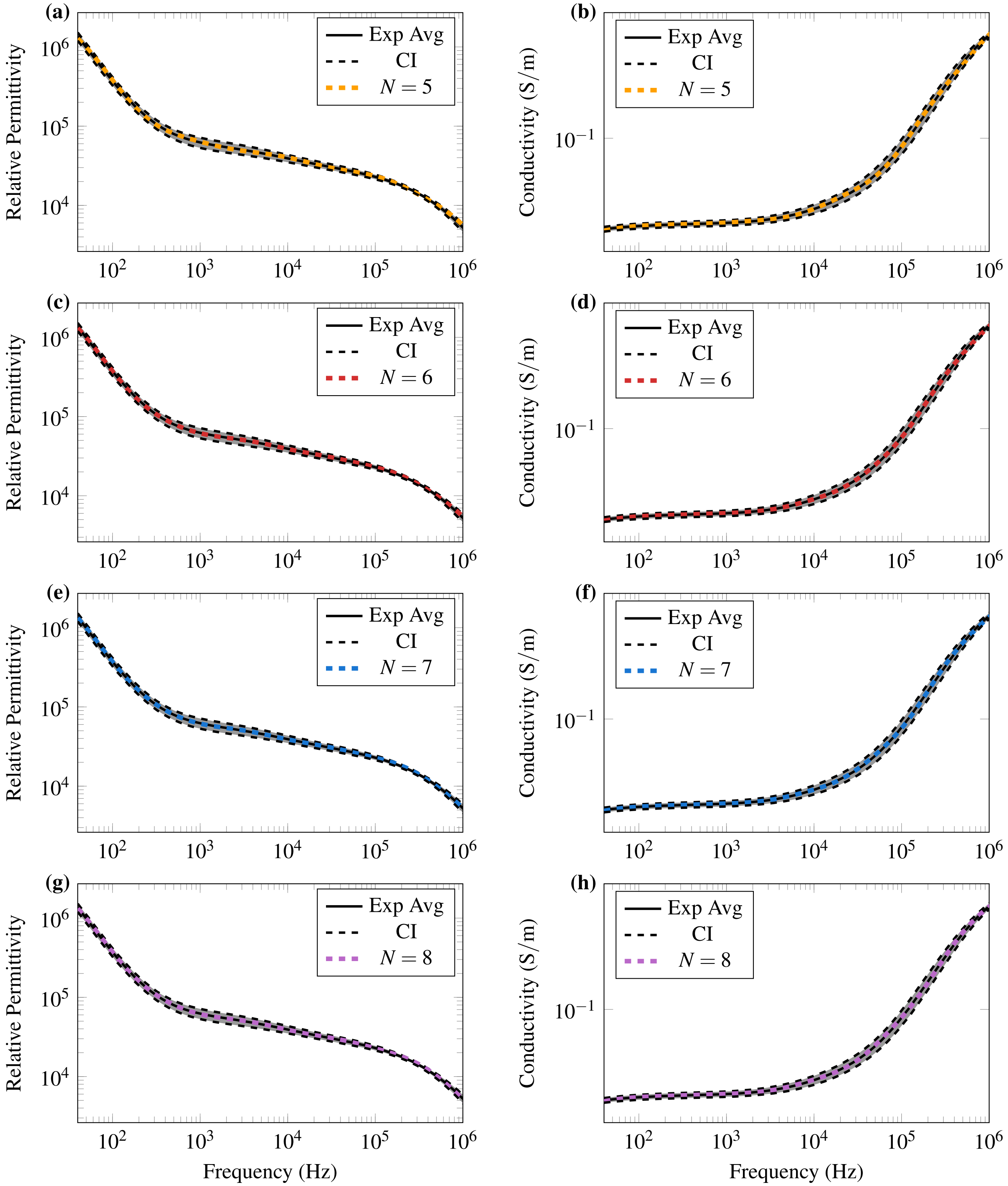

Figure S3. Experimental and parameterized results of **(a, c, e, g)** permittivity and **(b, d, f, h)** conductivity of potato tissue. Exp Avg is the experimental average, CI is the confidence interval (95%). N represents the number of Debye poles used to parameterize the experimental results. **(a, b)** 5 poles; **(c, d)** 6 poles; **(e, f)** 7 poles; **(g, h)** 8 poles.

S.4 Genetic Algorithm Code

```
% Experimental data load
experimental = readtable('directory_to_experimental_data');
experimental = table2array(experimental);

% -- Experimental data conditioning
frequency = experimental(:,1)';
experimental_cond = experimental(:,2)';
experimental_perm = experimental(:,3)';

%% Genetic Algorthim Code

% Number of poles of Debye dispersion
n_debye_poles = 6;

% Constant declaration
epsilon_0 = 8.854187817e-12;

% Frequency conversion
f = frequency;
w = 2*pi*f;

% Parameter Limits
delta_epsilon_upper_limit = 8;
delta_epsilon_lower_limit = -3;
tau_upper_limit = -1;
tau_lower_limit = -12;
epsilon_inf_upper_limit = 10; % Ideally inf, but 10 already works
epsilon_inf_lower_limit = 0;
sigma_s_upper_limit = 0;
sigma_s_lower_limit = -4;

% Multipole Debye dispersion model declaration
debye_func = @(epsilon_inf, sigma_s, delta_epsilon, tau)...
    10^epsilon_inf + 10^sigma_s./(1j*w*epsilon_0) + ...
    sum((10.^delta_epsilon)./(1 + (1j*w.*10.^tau)));

% Conversion of Experimental to Debye Real and Imaginary
experimental_real = experimental_perm;
experimental_imag = (-1)*experimental_cond./(epsilon_0*w);
experimental_func = experimental_real + 1i*experimental_imag;

% Number of variables of the Debye parameterization
n_variables = 2 + 2*n_debye_poles;

% Parameter limits vector initialization
lb = zeros(1, n_variables);
ub = zeros(1, n_variables,1);

% Parameter limits vectorization
% -- Lower limits
lb(1) = epsilon_inf_lower_limit;
lb(2) = sigma_s_lower_limit;
lb(3:(n_debye_poles + 2)) = delta_epsilon_lower_limit;
lb((n_debye_poles + 3):n_variables) = tau_lower_limit;
```

```
% -- Upper limits
ub(1) = epsilon_inf_upper_limit;
ub(2) = sigma_s_upper_limit;
ub(3:(n_debye_poles + 2)) = delta_epsilon_upper_limit;
ub((n_debye_poles + 3):n_variables) = tau_upper_limit;

% Genetic Algorithm declaration
ga_options_log = optimoptions('ga', ...
    'PlotFcn', @gaplotbestf2, ...
    'UseParallel', true, ...
    'MaxGenerations', 2e3,...
    'MaxStallGenerations', 500,...
    'PopulationSize', 1e3, ...
    'SelectionFcn', @selectiontournament, ...
    'MutationFcn', @mutationadaptfeasible);

% Vectorization of parameterization variables
% -- The 'p' vector is used in the next functions. The vector contains the
% -- results of the parameters, which are organized according to the
% -- following structure
% -- p(1) = epsilon_inf
% -- p(2) = sigma_s
% -- p(3) = delta_epsilon_1
% -- p(4) = delta_epsilon_2
% -- ...
% -- p(n_debye_poles + 2) = delta_epsilon_n
% -- p(n_debye_poles + 3) = tau_1
% -- p(n_debye_poles + 4) = tau_2
% -- ...

% Auxiliary function to handle the p vector
debye_to_fit = @(p)debye_func(p(1), p(2), p(3:(n_debye_poles + 2))',...
    p((n_debye_poles + 3):n_variables)');

% Cost function declaration
log_cf = @(p)sum(...
    (log10(real(experimental_func)) - log10(real(debye_to_fit(p)))).^2 + ...
    (log10(imag(experimental_func)) - log10(imag(debye_to_fit(p)))).^2 ...
);

% Parameterization process
[sol_log, fval_log] =  ...
    ga(log_cf, n_variables,[],[],[],[],lb,ub,[], ga_options_log);

% Generate Plot to visual inspection
% -- Extracting conducitivity and permittivity from the parameterized curve
cond_debye_solution = epsilon_0*(w).*imag(debye_to_fit(sol_log))*(-1);
perm_debye_solution = real(debye_to_fit(sol_log));
% -- Plot
figure(2);
subplot(2, 1, 1);
loglog(f,[experimental_perm; perm_debye_solution]);
subplot(2, 1, 2);
loglog(f,[experimental_cond; cond_debye_solution]);
```

```
% Print parameters
fprintf(’%s\n’,10^sol_log(1));
fprintf(’%s\n’,10^sol_log(2));
for i = 1:(n_debye_poles)
    fprintf(’%s\n’,10^sol_log(2 + i));
    fprintf(’%s\n’, 10^sol_log(n_debye_poles + 2 + i));
end

% Function to observe the CF during parametrization process
function state = gaplotbestf2(options,state,flag)
%GAPLOTBESTF Plots the best score and the mean score.
%   STATE = GAPLOTBESTF(OPTIONS,STATE,FLAG) plots the best score as well
%   as the mean of the scores.
%
%   Example:
%    Create an options structure that will use GAPLOTBESTF
%    as the plot function
%     options = optimoptions(’ga’,’PlotFcn’,@gaplotbestf);
%   Copyright 2003-2016 The MathWorks, Inc.
state.Score = real(state.Score);
if size(state.Score,2) > 1
    msg = getString(message(’globaloptim:gaplotcommon:PlotFcnUnavailable’,’gaplotbestf’));
    title(msg,’interp’,’none’);
    return;
end
switch flag
    case ’init’
        figure(1);
        hold on;
        set(gca,’xlim’,[0,options.MaxGenerations]);
        xlabel(’Generation’,’interp’,’none’);
        ylabel(’Fitness value’,’interp’,’none’);
        plotBest = plot(state.Generation,min(state.Score),’.k’);
        set(plotBest,’Tag’,’gaplotbestf’);
%         plotMean = plot(state.Generation,meanf(state.Score),’.b’);
%         set(plotMean,’Tag’,’gaplotmean’);
        title(’Best: ’,’interp’,’none’)
    case ’iter’
        best = min(state.Score);
%         m    = meanf(state.Score);
        plotBest = findobj(get(gca,’Children’),’Tag’,’gaplotbestf’);
%         plotMean = findobj(get(gca,’Children’),’Tag’,’gaplotmean’);
        newX = [get(plotBest,’Xdata’) state.Generation];
        newY = [get(plotBest,’Ydata’) best];
        set(plotBest,’Xdata’,newX, ’Ydata’,newY);
%         newY = [get(plotMean,’Ydata’) m];
%         set(plotMean,’Xdata’,newX, ’Ydata’,newY);
        set(get(gca,’Title’),’String’,sprintf(’Best: %g’,best));
    case ’done’
        LegnD = legend(’Best fitness’);
        set(LegnD,’FontSize’,8);
        hold off;
end
end
```
